# High Resolution, High Capacity, Spatial Specificity in Perceptual Learning

**DOI:** 10.3389/fpsyg.2012.00222

**Published:** 2012-07-25

**Authors:** Christophe C. Le Dantec, Aaron R. Seitz

**Affiliations:** ^1^Department of Psychology, University of California – RiversideRiverside, CA, USA

**Keywords:** double training, orientation discrimination, perceptual learning, spatial specificity, visual search

## Abstract

Research of perceptual learning has received significant interest due to findings that training on perceptual tasks can yield learning effects that are specific to the stimulus features of that task. However, recent studies have demonstrated that while training a single stimulus at a single location can yield a high-degree of stimulus specificity, training multiple features, or at multiple locations can reveal a broad transfer of learning to untrained features or stimulus locations. We devised a high resolution, high capacity, perceptual learning procedure with the goal of testing whether spatial specificity can be found in cases where observers are highly trained to discriminate stimuli in many different locations in the visual field. We found a surprising degree of location specific learning, where performance was significantly better when target stimuli were presented at 1 of the 24 trained locations compared to when they were placed in 1 of the 12 untrained locations. This result is particularly impressive given that untrained locations were within a couple degrees of visual angle of those that were trained. Given the large number of trained locations, the fact that the trained and untrained locations were interspersed, and the high-degree of spatial precision of the learning, we suggest that these results are difficult to account for using attention or decision strategies and instead suggest that learning may have taken place for each location separately in retinotopically organized visual cortex.

## Introduction

Research of perceptual learning has garnered a lot of attention in regard to classical findings showing a high-degree of specificity to the trained stimulus features. A number of early and influential studies found that training on perceptual tasks resulted in a striking degree of specificity to the features of the trained stimuli (Fiorentini and Berardi, 1980; Ball and Sekuler, 1982; Karni and Sagi, 1991). However, significant controversy has arisen regarding the extent to which training on perceptual tasks yields learning effects that are specific to the stimulus features of that task. In particular, a topic of key controversy is whether training at a particular location in the visual field transfers to performance at other visual field locations. Classically, such location specificity has been found in a variety of tasks, however, a number of recent studies have demonstrated that while training a single stimulus at a single location can yield a high-degree of stimulus specificity, that training multiple features, or at multiple locations (so-called double training; Xiao et al., 2008; Yu et al., 2010) reveals broad transfer of learning to untrained features or stimulus locations. These new results suggests that spatial specificity, which had previously been thought to be an indication of plasticity in retinotopic visual brain areas (Gilbert et al., 2001; Fahle, 2005), may be better accounted for by attention or by decision strategies to focus resources on a limited area of space for tasks that involve highly restricted stimulus regimes.

To address this controversy, we devised a high resolution, high capacity, perceptual learning procedure with the goal of testing whether spatial specificity can be found in cases where observers are highly trained to discriminate stimuli in many different locations in the visual field. We trained participants in a visual search task where stimuli were presented in concentric grids within a 16° diameter ring in which stimuli (target and distractors) could appear in 36 possible grid locations. Among these, 24 locations were randomly chosen as target locations during 9 days of training. During a post-test session, the 12 untrained locations were tested along with the 24 trained ones. An eye-tracker was used to implement a gaze-enabled display where the stimulus array only appeared when participants were fixating (this was important to make sure targets were consistently presented on the same retinotopic locations). We found a surprising degree of location specific learning, where performance was significantly better when target stimuli were presented at one of the 24 trained locations compared to when they were placed in one of the 12 untrained locations.

## Materials and Methods

### Overview

**Figure 1:**
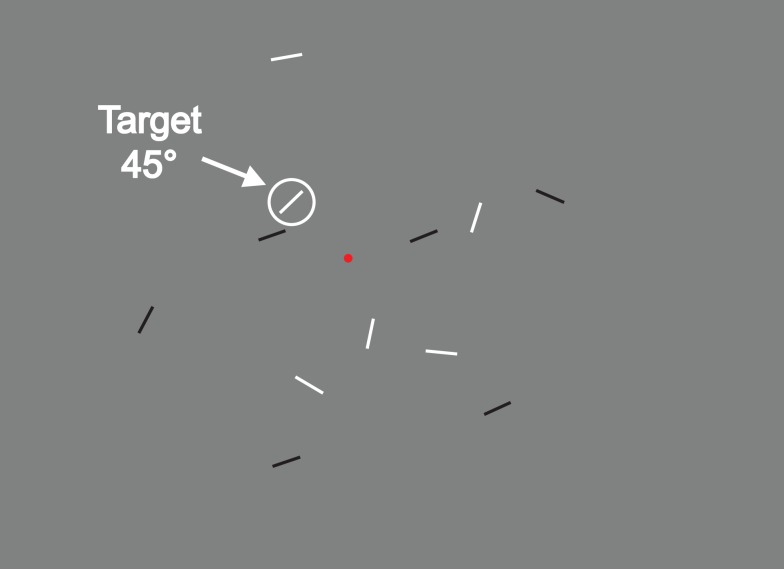
**Example of stimulus displays: here, the target oriented at 45°**. Participants had to fix the red fixation dot and, without moving their eyes to find the target and to determine if it is white or black.

During each trial, participants had to perform a visual search task (see Figure 1). They were required to fixate on a red dot located at the center of the screen and, while fixating, find among several short lines presented in periphery of the visual field, a pre-specified oriented target. As soon as they found the target, participants were required to indicate whether the color of the target was white or black via a key-press.

### Participants

Participants were nine undergraduate students (four females and five males; age range 19–25 years; mean 22.33 years, SD = 2.18 years) at the University of California – Riverside. They were all healthy, right-handed, and had normal or corrected-to-normal visual acuity. None of them reported any neurological, psychiatric disorders, or medical problems. Participants provided written informed consent at the beginning of the experiment and the experimental conditions conformed to the guidelines of the University of California – Riverside Human Research Review Board.

### Materials

An Apple^®^ Mac Mini running Matlab^®^ (MathWorks, Natick, MA, USA) and Psychtoolbox Version 3 (Brainard, 1997; Pelli, 1997) were used to generate the stimuli and control the experiment. Participants sat on a height adjustable chair at 50′′ to 55′′ inches from a 24′′ Sony Trinitron^®^ CRT monitor (resolution: 1600 × 1200 at 100 Hz). Gaze position on the screen was tracked with the use of an eye-tracker (EyeLink 1000^®^, SR Research).

### Stimuli

**Figure 2:**
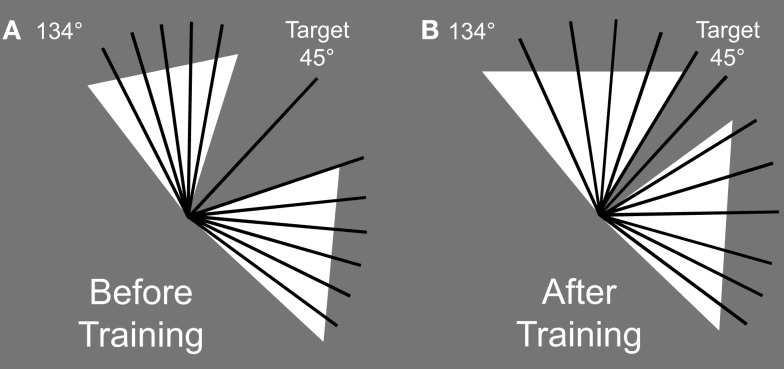
**Example of distractor orientation sets (dotted black lines) and the target (here target orientation = 45°, solid black line) at the beginning of training (A) and at the end of training (B)**. The white triangles represent the extent of the possible orientations of the distractors.

The stimuli were bright (95 cd/m^2^) or dark (5.5 cd/m^2^) lines (0.1° × 1°) presented on a gray (40 cd/m^2^) background (Figure 1). Participants were trained to find a target with an orientation (135° or 45°, counterbalanced across participants) among a set of distractors (ranging from 316° to 44° or 46° to 134°) and report whether the target was white or black (randomized across trials). The distractor range (an orientation wedge centered on 0° or 90°) was determined with a staircase procedure (see below for description) such that the closest distractor orientation of the wedge was adaptively moved as close to the target orientation as the participant could tolerate and perform well. For a given trial, the range of distractor orientations was chosen uniformly across the extent of the wedge such that there was always one distractor orientation present at the threshold value (see Figure 2). During the testing session, participants ran the same task but with trained and untrained target orientations.

**Figure 3:**
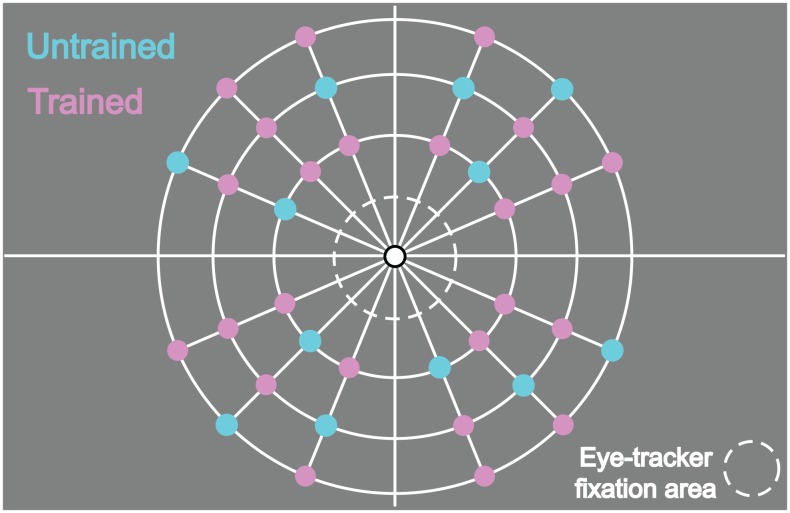
**Grid indicating the 36 possible positions (9 in each quadrant) for the target and the distractors**. During each trial, 12 stimuli (always 1 target and 11 distractors) were presented at the same time (3 in each quadrant). For each participant, 24 locations were chosen to serve as trained locations for the target during the training. Then, 12 other locations were used as untrained locations for the target during the test sessions. A different set of locations was employed for each participant.

The spatial locations of targets and distractors were presented on a grid (see Figure 3) such that the eccentricity (3°, 5°, 8°), and placement in the left/right and upper/lower visual quadrants was balanced across stimuli. Each line could be presented in one of nine locations (three at each eccentricity; 16.875°, 45°, 73.125° from the cardinal axis) in each quadrant. Three lines were presented in each visual quadrant for a set size of 12 search items. To prevent the occurrence of displays where all items were presented at the same eccentricity, we added the further constraint that all displays contained at least three items in each eccentricity. We pre-calculated all possible configurations of the 12 items within the grid given the above constraints. From this set of possible search displays each configuration was presented only once during the whole experiment.

Stimuli were presented in concentric grids within a 16° diameter ring in which stimuli (targets and distractors) could appear in 36 possible grid locations (see Figure 3). For each participant, we chose 24 locations to be used as target locations during the training phases. These were selected randomly with the constraint that the remaining 12 locations were matched in eccentricity to one of the trained target locations and were equally distributed across the four visual quadrants.

### Procedure

For all participants and all sessions, a gaze contingent display was utilized such that the participant had to fixate on a centrally presented red dot for 500 ms in order for each trial to begin. This was controlled by the use of the eye-tracker. Then, the search display was presented for 100 ms followed by a gray screen to which the participant had 2000 ms to indicate the color of the target with a key-press (“1” for white or “2” for black). Trials were determined to be invalid if an eye-movement was made while the search display was on the screen, no response was given, or a key other than “1” or “2” was pressed.

The experiment was divided into five phases. Each session and each phase was conducted on separate days. Sessions were mostly conducted on subsequent days, however, in some cases there was a 1- or 2-day gap between sessions (typically during the weekend).

In phase 1, participants were given detailed instructions and ran 20 practice trials to familiarize themselves with the task. The practice trials were completed with feedback. According to their response, the words “correct” or “wrong” were respectively presented at the center of the screen for 1 s. Then, the next trial started by the presentation of a red fixation dot.

In phases 2 and 4, sessions were conducted that were similar to those used in the test sessions (described below) and included both the trained and untrained target orientations but only at the trained locations. During this session, EEG recordings (not reported in this paper) were undertaken.

In phase 3 (Training phase), participants were trained on the visual search task during eight successive days. Each session consisted of 1000 trials that were split into eight blocks with a short break between blocks, and lasted approximately 1 h. Each session followed the general procedure described above and started with 20 practice trials, each followed by a visual feedback. For the remaining trials of each session, no feedback was given to the participants. During this phase, the targets were presented only at the trained locations.

Training sessions were organized into miniblocks. After each miniblock, the orientation range of the distractors was adjusted with a staircase procedure such that the distractor range was increased if the average performance of the previous block was greater than 80% correct; the range was decreased if the previous block performance was lower than 70% correct. The value for the new block was set to the current threshold value (orientation difference of target and closest distractor) multiplied by the difference between the proportion correct for that block and 0.75. This procedure was based upon pilot experiments that found stable threshold estimates and asymptotes using this procedure with the present task and stimuli. Threshold values reported in the manuscript represent the performance in the final miniblock of a given session. Data for each participant and each session was visually inspected to ensure that these values were stable and represented valid threshold estimates.

In phase 5, participants conducted two test sessions on two different days. Each session lasted approximately 1 h and started with 20 practice trials each followed by a visual feedback. For the remaining trials of each session, no feedback was given to the participants. The sessions followed the general procedure described above, except the participants had to run the task with trained and untrained target orientations in separate interleaved blocks. For these tests, a separate staircase (blockwise procedure identical to that used during training) was run on each of the orientation conditions. In test session 1, target locations were tested at the 24 trained and 12 untrained locations on the grid. In session 2, the 24 trained locations were again tested, but untrained locations were selected from a slightly different grid (eccentricities 4°, 6.5°, 9° and at 0°, 30°, 60° from the cardinal axes) so that novel target locations were employed.

### Analyses

Data were analyzed as appropriate to a within-participant design. In all cases repeated measures ANOVAs and paired *t*-tests were utilized. Also error bars in figures are within-participant error bars (Loftus and Masson, 1994).

## Results

**Figure 4:**
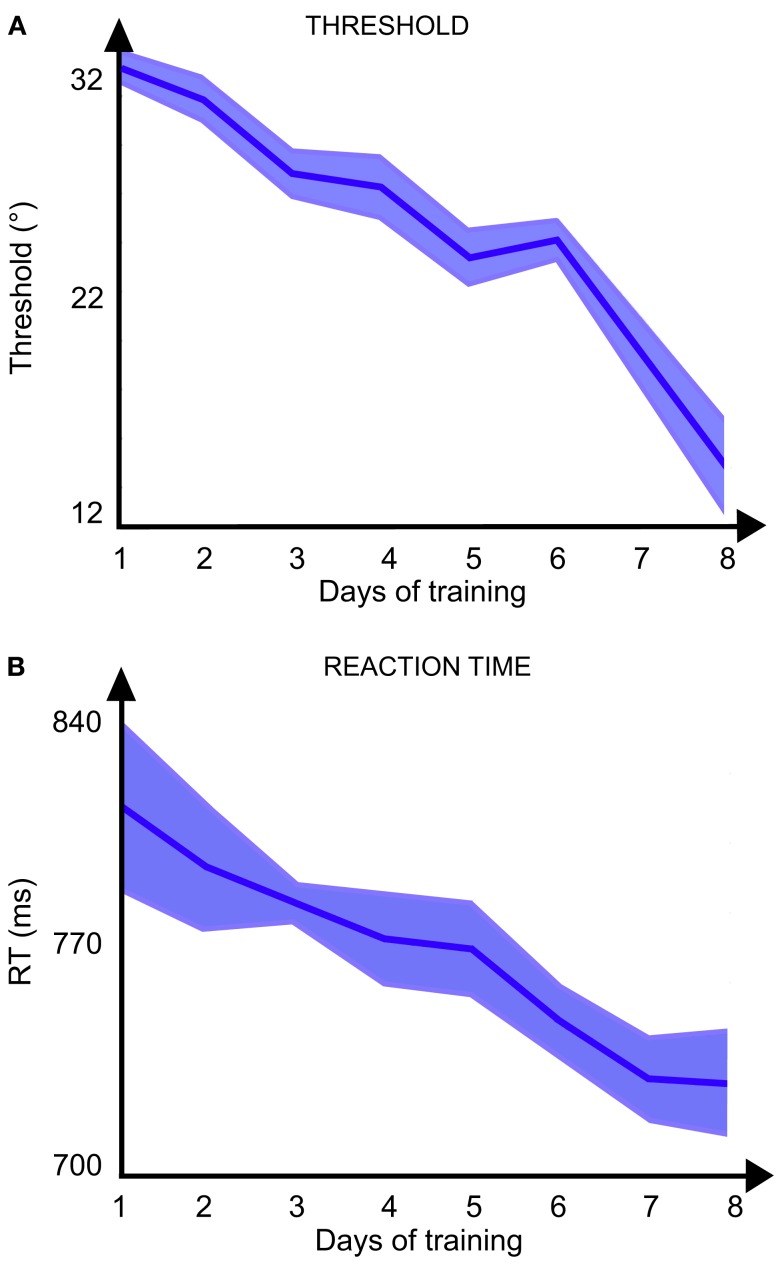
**Results of training sessions: (A) threshold (distance in° between the orientation of the target and the possible orientation of the distractors) and (B) reaction time as a function of the day of training**.

Data from the training sessions showed a significant effect of Perceptual Learning. This learning can be seen in Figure 4 where the smallest orientation difference that the participants could discriminate [from 31.2° ± 0.6° to 14.3° ± 2.0° *F*(7, 8) = 3.29; *p* = 0.0053 ANOVA] decreased across sessions as the reaction time also decreased [from 811.4 ± 25.3 to 730.8 ± 17.7 ms; *F*(7, 9) = 7.38, *p* < 0.0001 ANOVA]. The evolution of the performance shows that the participants became more and more efficient in the task as the training progressed.

**Figure 5:**
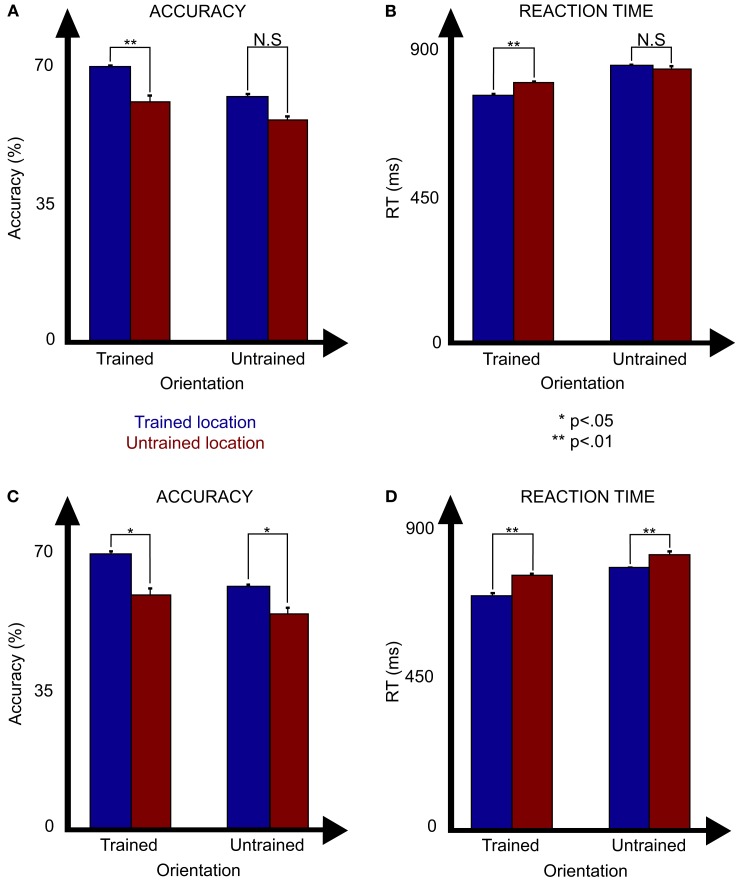
**Results of tests session 1 [(A) accuracy and (B) reaction time] and 2 [(C) accuracy and (D) reaction time] as a function of the orientation (trained or untrained) and the location of the target (trained or untrained)**.

The testing sessions were designed to assess the specificity of learning to the trained locations. To accomplish this, performance on a set of 12 untrained target locations was compared to that of the 24 trained locations. During the first test session, as indicated in Figure 5A, the trained target presented in the trained location produced significantly more accurate responses [*F*(1, 8) = 12.8; *p* = 0.0071 ANOVA], with significantly higher accuracy at the trained location for the trained orientation (70.9 ± 4.8 vs 62.3 ± 6%; *p* = 0.0021 *t*-test) and also for the untrained orientation (63.2 ± 2.5 vs 57.3 ± 4.2%; *p* = 0.0036 *t*-test). Response time data tells a similar story (Figure 5B) with significantly faster responses at the trained locations than the untrained ones [*F*(1, 8) = 27.3; *p* = 0.0008 ANOVA] and significantly faster responses at the trained location for the trained orientation (768.7 ± 26.2 vs 809.1 ± 24.0 ms; *p* = 0.0039 *t*-test), however not for the untrained orientation (863.7 ± 24.7 vs 854.1 ± 35.9 ms; *p* = 0.55 *t*-test). There was also a significant difference between the trained and untrained orientations for accuracy [*F*(1, 8) = 76.3; *p* < 0.0001 ANOVA] and marginally so for response times [*F*(1, 8) = 36.3; *p* = 0.093 ANOVA], with a significant interaction between orientation and location for response times [*F*(1, 8) = 5.9; *p* = 0.042 ANOVA] but not accuracy [*F*(1, 8) = 0.75; *p* = 0.41 ANOVA]. Of note, the degree of location specificity found for the untrained orientation, while weaker than that for the trained location, likely resulted from learning produced through the multiple sessions that participants conducted with the untrained orientation prior to this test.

These data clearly show that there is a performance advantage for the trained locations compared to the untrained ones. However, this could be because of an enhancement of performance for the trained locations or a suppression of performance for the untrained ones. For example, at trained locations, participants either experienced targets or distractors, however at untrained locations, only distractors were presented. This could have led to a suppression in processing the untrained locations (e.g., negative priming; e.g., Tipper, 1985). To control for this possibility, we ran a second test in which untrained target locations were selected from locations in-between the trained grid such that at these locations neither targets nor distractors had been presented during training. If the poorer performance for the untrained target locations was due to poorer performance at locations where distractors were present, then even better performance would result for untrained compared to trained locations. However, this was not the case. Similar to the first test (Figure 5C), the trained locations produced significantly more accurate responses [*F*(1, 5) = 8.0; *p* = 0.038 ANOVA] with significantly higher accuracy at the trained location for the trained orientation (71.4 ± 2.9 vs 60.1 ± 5.4%; *p* = 0.01 *t*-test) and the untrained orientation (63.0 ± 2.0 vs 56.0 ± 4.2%; *p* = 0.014 *t*-test). Response time data (Figure 5D) tells a similar story with significantly faster responses at the trained locations than the untrained ones [*F*(1, 5) = 14.5; *p* = 0.007 ANOVA] and significantly faster responses at the trained location for both the trained orientation (733.2 ± 39.4 vs 798.0 ± 20.2 ms; *p* = 0.018 *t*-test) and untrained orientation (821.0 ± 17.1 vs 864.3 ± 37.0 ms; *p* = 0.041 *t*-test). There was also a significant difference between the trained and untrained orientations for accuracy [*F*(1, 5) = 122.2; *p* < 0.0001 ANOVA] and for response times [*F*(1, 5) = 11.3; *p* = 0.02 ANOVA], without significant interactions between orientation and location for response times [*F*(1, 5) = 2.7; *p* = 0.16 ANOVA] or accuracy [*F*(1, 5) = 0.66; *p* = 0.45 ANOVA]. These results confirm the first findings of location specificity and suggest that this is due to the enhancement of the trained locations rather than the suppression of the untrained locations.

**Figure 6:**
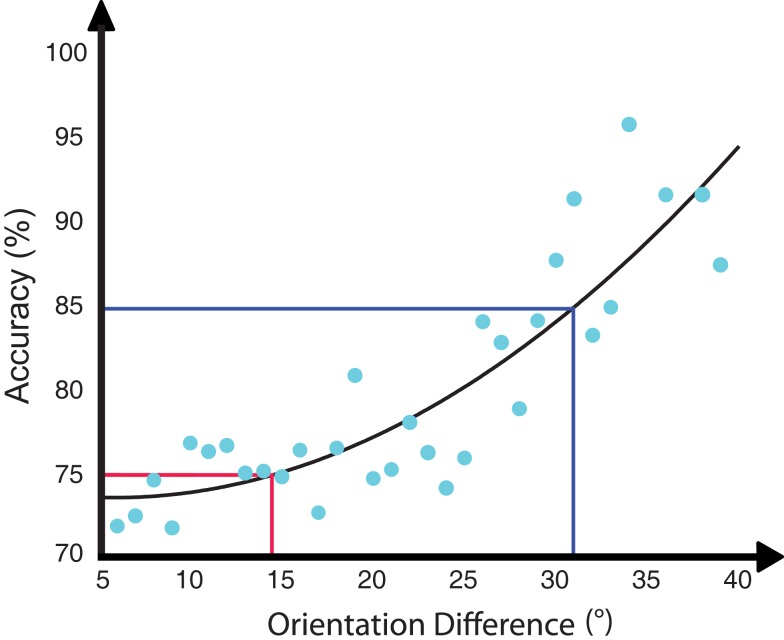
**Participant average psychometric function calculated from the last training session**. Each dot represents performance from one block for one participant. The performance change from the psychometric function suggests that the extent of transfer to untrained location was minimal.

While our data clearly demonstrates location specificity of learning, there is an outlying question of how much of the learning may have transferred across locations. Specifically, how can we relate the change in threshold of 16.9° found across the training sessions to the 8.6 difference in accuracy found between the trained and untrained locations for the trained orientation? To address this question, we examined the staircase data from the last training session. Given that the staircase was run in a block wise manner we were able to calculate performance for each block for each participant and estimate the participant average psychometric function (see Figure 6). From this, we first calculated a linear fit and estimated a slope of approximately 0.55 change of accuracy per degree. Using this slope, we calculated that the threshold change observed during training would map on to a change of accuracy of 9.3. As a second analysis, we fit a quadratic function to the data and from this calculated a change of accuracy of 10.2. Thus, while we lack a direct measure of the thresholds at the untrained location, our estimates of the performance change from the psychometric function suggests that the extent of transfer to the untrained location was minimal.

## Discussion

The degree to which training on perceptual tasks is specific to the trained stimulus features has been of key interest in studies of perceptual learning. For example, Fiorentini and Berardi (1980) trained participants to discriminate oriented gratings of different luminance distributions and noted that the trained benefits failed to transfer to similar stimuli but with untrained orientations and spatial frequencies. Later, Poggio et al. (1992) found that improvements of Vernier discrimination were specific to the trained location, angle of orientation, and even to the eye that viewed the stimuli during training. They conjectured, on computational grounds and knowledge of the functional architecture of the visual system, that this was a result of low-level visual plasticity. Correspondingly, a number of electrophysiological studies found signs of plasticity in low-level visual brain areas, including V1 (Crist et al., 2001; Schoups et al., 2001), V4 (Yang and Maunsell, 2004; Raiguel et al., 2006; Franko et al., 2010), MT (Zohary et al., 1994), among others, and the view that specificity of Perceptual Learning implied the locus of brain plasticity became quite influential (Gilbert et al., 2001; Fahle, 2004).

However, the interpretation of behavioral findings of specificity has been questioned on theoretical grounds with models showing that the visual system can exhibit stimulus-specific learning effects even when allowing no plasticity within the parts of the model engaged in perceptual processing (Dosher and Lu, 1998; Sotiropoulos et al., 2011). For example, in an influential model by Dosher and Lu (1998), plasticity in the read-out – weights between the representational and decision areas – well accounts for many observed perceptual learning effects and argues against the sufficiency of stimulus-specific learning effects as evidence for plasticity in visual brain areas. Accordingly, a number of influential electrophysiological studies have failed to find plasticity in visual brain regions that were known to be involved in processing the trained stimulus features (Crist et al., 2001; Ghose et al., 2002; Law and Gold, 2008; Gu et al., 2011).

Recent behavioral studies further challenge the postulate that stimulus-specific learning results from low-level visual processing. Application of the recently developed technique of “double training” found that the specific learning effects found in their paradigms can show broad transfer when more than one stimulus attribute is trained at a time. Xiao et al. (2008) trained participants on Vernier discrimination task at a specific orientation at a specific location in the visual field, which normally yields location and orientation specific learning effects (Poggio et al., 1992). But when they subsequently trained a second orientation at a different spatial location, they found that the training induced changes for the second orientation transferred to the first location. Such findings of broad location transfer undermine the argument that this learning is due to plasticity in retinotopic visual areas.

The present study addresses this controversy by examining the extent to which location specificity can be found when a given perceptual feature is trained at many locations. We employed a visual search task where untrained locations were highly proximal to and interspersed with the trained locations. This can be considered an extreme case of double training, where not 2, but 24 visual field locations were trained. Still, even with 24 times the training, we found that post-training performance was significantly higher in trained locations compared to untrained locations that were just a couple degrees of visual angle away from those that were trained. We think that the key to our findings of specific perceptual learning is the fact that we trained participants on fine-orientation discrimination, similar to those which have resulted in plasticity in visual cortex (Schoups et al., 2001), and trained participants using a gaze-enabled display where eye-movements were tightly monitored and stimuli were only displayed when participants were looking at a fixation point in the center of the display. The use of this fine-control of eye-movements is important because eye-movements in these tasks can result in inadvertent stimulation of visual field locations as participants move their eyes around the display.

Notably, while we observed both location and orientation specificity in our study, we did not consistently find an interaction between the two. As can be seen in Figure 5, performance at the untrained location was consistently more accurate and faster for the trained compared to the untrained orientation. This aspect of the results is consistent with those of double training studies (Xiao et al., 2008; Yu et al., 2010) where there is some degree of orientation specific learning that transfers to untrained locations. However, training induced effects of orientation were stronger at the trained location and performance at both orientations was better at the trained location. Overall, our results suggest that while there is some degree of transfer to untrained locations, this transfer is incomplete, as claimed by Xiao et al. (2008).

There exist a growing number of studies that address how specificity, or its opposite, transfer, is controlled by different factors. In a discrimination task, Jeter et al. (2009) showed that transfer was observed in low-precision transfer tasks while specificity was observed in high-precision transfer tasks. Then, Jeter et al. (2010) showed that specificity was the result of an extensive training, confirming more classical results (Fiorentini and Berardi, 1980; Ball and Sekuler, 1982; Karni and Sagi, 1991), while a substantial transfer was observed at early in the training. Interestingly, another study, reported by Aberg et al. (2009) presented a series of experiments showing, on one hand, that the number of trials per session influenced the overall improvement of the participant’s performance, and on the other hand, the transfer depended on the number of trials presented during each session, not the total number of trials. Zhang et al. (2010) showed the peripheral orientation discrimination tasks transferred to new locations only after a pre-test was given to participants. These studies add to the double-training studies that show transfer after training multiple features or at multiple locations (Xiao et al., 2008; Yu et al., 2010). Together these studies show that many factors (extent of training, blocking of trials, precision of training stimuli, diversity of training set, etc.) influence the transfer of learning. Further research will be necessarily to see how these factors interact and modeling is needed to make predictions regarding the expected level of specificity/transfer for a given experimental set.

Could the lack of transfer in our task be related to the fact that we didn’t include a pre-test where performance at all 36 locations was assessed? Highlighting this issue is the above mentioned study by Zhang et al. (2010), which found that the inclusion of a pre-test was enough to enable training on fine-orientation discrimination to transfer across locations. This is consistent with their rule-based learning framework (Yu et al., 2010) explaining how training multiple features encourages decision processes to generalize. In fact, recent research into this rule-based learning shows that training at just two locations (Zhang et al., 2011) is sufficient to unlock transfer across the visual field. However, in our case, we had a pre-test for the two orientations that were used and 24 of the 36 locations that were evaluated. What we lacked was a pre-test for the 12 novel locations, which were proximal and interspaced with the trained locations. This demonstrates either that the learning was retinotopically specific or that these learned decision rules are highly specific to the trained locations. Either way the high-degree of location specificity observed in our study is notable.

A key question regards why broad spatial transfer was not found in our study but was found in previous research using double training approaches. One difference may be due to the choice of stimuli employed. A visual search paradigm was used in our studies where relatively large orientation differences (15–30° of visual angle) existed between the target and the distractors. While double training was employed with a similar configuration in Yu et al. (2010), their paper concentrated on orientation and did not address whether spatial transfer occurred in that experimental configuration. On the other hand, Xiao et al. (2008) used a fine-orientation discrimination task and found spatial transfer. A number of studies have noted different mechanisms of perceptual learning between fine and coarse orientation discrimination tasks (Butts and Goldman, 2006; Raiguel et al., 2006; Adab and Vogels, 2011) and this could result in different transfer effects from double training. Another difference in our study regards the complexity of the stimulus judgment; picking out a target orientation that can appear at one of a large number of target locations from a set of differently oriented distractors that also appear at scattered spatial locations, which according to the reverse hierarchy theory of perceptual learning, would promote learning at a relatively early stage of visual processing (Ahissar and Hochstein, 1997). Finally, the difference between studies may depend upon the larger number of locations trained in our study and their proximal location to the untrained locations. This may have resulted in interference (Seitz et al., 2005) of processing at the untrained locations. Future studies will need to be conducted to better address these issues and to clarify mechanisms involved in these studies.

Our results are important because they show that location specificity can be found, even when multiple locations are trained. Given the large number of trained locations, the fact that the trained and untrained locations were interspersed, and the high-degree of spatial precision of the learning, we suggest that these results pose a challenge to accounts of perceptual learning based upon attention or decision strategies and instead imply that learning may have taken place for each location separately in retinotopically organized visual cortex. These results suggest that the extent of specificity may depend highly upon the details of the training procedure and stimulus set and that training multiple stimulus locations does not necessarily result in transfer to untrained locations.

## Conflict of Interest Statement

The authors declare that the research was conducted in the absence of any commercial or financial relationships that could be construed as a potential conflict of interest.
